# Comparing Mindfulness Based Cognitive Therapy and Traditional Cognitive Behavior Therapy With Treatments as Usual on Reduction of Major Depressive Disorder Symptoms

**DOI:** 10.5812/ircmj.8018

**Published:** 2013-02-05

**Authors:** Abdollah Omidi, Parvaneh Mohammadkhani, Abolfazl Mohammadi, Fatemeh Zargar

**Affiliations:** 1 Department of Clinical Psychology, Kashan University of Medical Sciences, Kashan, IR Iran; 2 Department of Clinical Psychology, University of Social Welfare and Rehabilitation Sciences, Tehran, IR Iran

**Keywords:** Cognitive Behavior Therapy, Major depressive Disorder, psychiatric

## Abstract

**Background:**

In this studyMindfulness and CBT were combined to investigate the enhance of psychotropic work. Both therapies have integrated acceptance-based mindfulness approaches with change-based cognitive behavioral therapies to create efficacious treatments. That is, introduce use of MBCT in active phase of treatment and chronic depression.

**Objectives:**

This study was done to evaluate efficacy of Mindfulness Based Cognitive Therapy (MBCT) and traditional Cognitive Behavior Therapy (CBT) with Treatments as usual (TAU) to reduce psychiatric symptoms in a sample of patients with Major Depressive Disorder (MDD).

**Materials and Methods:**

90 patients who were referred to clinics of university of Social Welfare and Rehabilitation Sciences and Tehran University Counseling Centre and met DSM-IV criteria for MDD were selected. They were randomly assigned to MBCT (n = 30), CBT (n = 30), or TAU (n = 30). They were aged between 18 and 45 years (M = 28, SD = 8), with an average of two previous depression episodes. They were interviewed through the Structured Clinical Interview for DSM-IV and self-report by Brief Symptom Inventory, pre and post treatment. Patients in MBCT and CBT group received the treatment, while TAU group continued therapy (anti-depressant).

**Results:**

The results indicated that MBCT and CBT groups have significant efficacy on reduction of MDD symptoms.

**Conclusions:**

MBCT appears to be as effective as CBT in the treatment of current depression.

## 1. Background

The emotional, social and economic burden of depression for suffers, their families and society is significant, with 12 month prevalence rates estimated at 2.9-12.6% and lifetime risk estimated at 17-19% (1). The fact that depression is often a chronic relapsing and pervasive condition, with relapse rates of 50-80% in those who have been depressed before (2) has contributed to the WHO prediction that, by 2020, depression will be the second biggest contributor to ill-health burden world-wide (1). These patients report continuing symptoms of depression and accompanying distress about these symptoms. Nolen-Hoeksema's Responses Styles Theory (1991) suggests that people who engage in "repetitive and passive thinking about one's symptoms of depression "tend to prolong the very symptoms they are trying to reduce. Ruminators often hold positive (but erroneous) beliefs that it will help, not realizing that they are reducing their capacity to effectively problem- solving (3). Of particular concern is the risk of suicidal behavior in such patients. One in seven patients hospitalized for major depressive disorder die by suicide (4) and increase this rate associate with psychological distress and accompanying symptoms. Evidence suggests that the Major Depressive Disorder has many symptoms and disorders co-morbid that maintain depression (5-8). Some of these symptoms and disorders are anxiety, phobia, somatization, paranoia, aggression, interpersonal sensitivity, and obsessive compulsive disorder (1).

CBT has been shown to be effective at treating acute depression, but prophylactic effects of that is controversial (8). CBT for depression involves the application of specific, empirically supported strategies focused on cognition and behavior (2). CBT has generally been associated with a lower degree of relapse than those for medication. Although methodologies vary significantly between studies, figures suggest that 0-50% of patients exhibit symptom relapse or recurrence within 1-2 years of discontinuing cognitive behavioral treatment (9).

Mindfulness-based Cognitive Therapy (MBCT) is a class-based program designed for use in the prevention of relapse of major depression. Its aim is to teach participants to disengage from those cognitive processes that may render them vulnerable to future episodes. Mindfulness is commonly defined as the state of being attentive to and aware of what is taking place in the present (10). Although the mechanism through which mindfulness enhances psychological and behavioral functioning remains unclear (10), researches indicate that the enhancement of mindfulness is associated with a variety of well-being outcomes such as reduced pain (11, 12), anxiety, depression, binge eating (13), and stress (14). Mindfulness captures a quality of consciousness that is characterized by clarity and vividness of current experience and functioning and thus stands in contrast to mindless, less “awake” states of habitual or automatic functioning that may be chronic for many individuals. Mindfulness may important in disengaging individuals from automatic thoughts, habits, and unhealthy behavior patterns and thus could play a key role in fostering informed and self-endorsed behavioral regulation, which has long been associated with well-being enhancement (15).

Further, by adding clarity and vividness to experience, mindfulness may also contribute to well-being and happiness in a direct way. Many theories in psychopathology and psychotherapy have discussed the importance of observant, awareness and attention in the optimization of well-being (16, 17). Thus emphasizes changing the awareness of, and relation to, thoughts, rather than changing thought content. MBCT offers participants a different way of being with emotional pain and distress. The assumption is that cultivating a decentered relation to negative thinking provides one with the skills to prevent escalation of negative thinking at times of potential relapses (2). Mindfulness practice invites the mediator to notice and accept this thought as an event occurring in the mind rather than as a truth that defines the self. Thus mindfulness can alter one's attitude or relation to thoughts, such that they are less likely to influence subsequent feelings and behaviors. In contrast, CBT involves the restructuring and disputation of cognitions and beliefs toward acquiring more functional ways of viewing the world (18). Mindfulness and CBT were combined to investigate the enhance of psychotropic work. Both therapies have integrated acceptance-based mindfulness approaches with change-based cognitive behavioral therapies to create efficacious treatments. That is, introduce use of MBCT in active phase of treatment and chronic depression.

## 2. Objectives

This study was done to evaluate efficacy of Mindfulness Based Cognitive Therapy (MBCT) and traditional Cognitive Behavior Therapy (CBT) with Treatments as usual (TAU) to reduce psychiatric symptoms in a sample of patients with Major Depressive Disorder (MDD).

## 3. Materials and Methods

### 3.1. Participants and Procedure

A randomized clinical trial design was used. Ninety patients were recruited from the clinical services of the University of Social Welfare and Rehabilitation Sciences, and Tehran University Counseling Centre. To take part in the study, participants needed to have a record of major depressive disorder meeting DSM-IV criteria. Each participant was visited for verification, agreement, and written permission to assess the patient for the study. The inclusion criteria for entry were that participants had to meet DSM-IV criterion for MDD. And the exclusion criteria were that is they BMD, psychosis, drug abuse, organic history, eating disorder, and suicidal patients.

Participants were selected randomly, and allocated to three equal groups (30 for MBCT, 30 for CBT and 30 for TAU). They were aged between 18 and 45 years, 30 were male and 60 were female, all having been treated by psychiatrist before the study. All patients remained under outpatient care during the study. Assessment of clinical state were made pre and post treatments.

The treatment methods used in this study were MBCT, CBT and TAU. The course followed the MBCT program as described by Segal, Williams, and Teasdale (18), that translated in Persian by Mohammadkhani and Rezaee Dogaheh (19). Eight 2-h classes were held, up to an hour of which is spent in meditation practices. Home work involved around 1 h per day of meditation or behavioral and other related formal and informal practices for the eight weeks. Besides, behavioral enhancement components of CBT for depression were integrated (19). The structure and format of cognitive behavior therapy closely followed that of the original 8-week CBT course, described by Emery (2000), that translated in Persian by Mohammadkhani and Rezaee Dogaheh (19). TAU patients continued under the care their any therapy. (anti-depressant medication by psychiatrist). The treatment for depression actually received by patients in the TAU condition was monitored over the intervention period and is summarized in Table 1. The corresponding data for patients in the other groups are also shown for comparison. There were no statistically significant differences between the TAU and the others conditions.

**Table 1.** Means and Standard Deviations for the BSI Scales for MBCT, CBT , and TAU Groups at Pre-test and Post-test

	MBCT		CBT		TAU	
	Pre-test	Post-test	Pre-test	Post-test	Pre-test	Post-test
**GSI**	1.62 (0.56)	0.72 (0.46)	1.79 (0.47)	0.72 (0.26)	1.79 (0.50)	1.59 (0.52)
**Somatization**	1.70 (0.68)	0.74 (0.61)	1.71 (0.79)	0.75 (0.45)	1.66 (0.77)	1.74 (0.76)
**OCD**	1.90 (0.74)	0.96 (0.69)	1.91 (0.77)	0.91 (0.45)	2.07 (0.76)	1.94 (0.75)
**Interpersonal Sensitivity**	1.62 (0.70)	0.73 (0.49)	2.07 (0.97)	0.69 (0.38)	1.58 (0.92)	1.85 (0.86)
**Depression**	2.05 (0.84)	0.79 (0.63)	2.18 (0.57)	0.79 (0.51)	2.18 (0.85)	1.96 (0.86)
**Anxiety**	1.83 (0.81)	0.92 (0.73)	2.11 (0.82)	0.77 (0.49)	1.75 (0.90)	1.58 (0.78)
**Aggression**	1.85 (0.70)	0.52 (0.76)	1.90 (0.67)	0.71 (0.36)	1.98 (0.72)	1.62 (0.78)
**Phobia**	1.12 (0.83)	0.37 (0.48)	1.65 (0.85)	0.50 (0.30)	1.51 (0.66)	1.58 (0.77)
**Paranoia**	1.74 (0.80)	0.81 (0.54)	1.45 (0.93)	0.85 (0.45)	1.61 (0.92)	1.71 (0.79)
**Psychosis**	1.84 (0.89)	0.85 (0.56)	1.67 (0.73)	0.74 (0.46)	2.03 (0.80)	1.44 (0.82)

### 3.2. Measures

#### 3.2.1. Structured Clinical Interview for DSMIV Axis I Disorders SCID-I (SCID-I/CV)

Structured Clinical Interview for DSMIV Axis I disorders SCID-I (SCID-I/CV) is a comprehensive and standardized instrument for assessing major mental disorders in clinical and research atmosphere (20). SCID-I is administrated in a single session and takes about 45 to 90 minutes. The validity and reliability of this instrument have been confirmed in the different studies (21). Zanarini et al (22) has been reported inter-rater diagnostic reliability with Kappa upper than 0.7 in the most diagnoses. Persian version of this questionnaire has been provided by Sharifi et al. (23). The validity of the instrument has been confirmed by clinical psychologists and its retest reliability was 0.95 for one week.

#### 3.2.2. Brief Symptom Inventory (BSI)

The was used to assess psychiatric symptoms because of brevity, sensitivity to change , and well- documented reliability and validity (24). Each of its 53 items is rated on a 5-point Likert–type scale .The General Severity Index, a weighted frequency score based on the sum of the rating of all items, was useful as a measure of current symptoms. This index has a reported of 0.85 (25), and the coefficient was 0.89.

### 3.3. Data Analysis

To confirm symptomatic differences between the MBCT, CBT and TAU groups, analysis of co-variance (ANCOVA) was used to investigate changes between pre and post intervention scores on the total scales of BSI.

## 4. Results

Ninety individuals were studied before and after completion of the MBCT, CBT, TAU (30 for per group). All reported analysis examines the samples. Mean and standard deviations for the intake variables are presented in Table 2. There were no differences between the completers and non-completers on any of the demographic variables, severity of psychiatric states, diagnostic criteria, so the primary analysis was conducted on the entire treated and non-treated samples. Means and standard deviations are shown in Table 1.

**Table 2.** Baseline characteristics of treatment MBCT, CBT, and Treatment as Usual (TAU)

	MBCT	CBT	TAU
**Female (%)**	80	66	53
**Age (years)**	32 ± 6.3	30 ± 5.2	35 ± 4.8
**Marital status (%)**			
Single	26	20	25
Married	68	70	65
Separated	6	10	10
**Years of education**	12.5±4.5	12± 2.5	12± 4
**Antidepressant medication**	100	100	100
**Psychotherapy/counseling**	34	39	38

Analysis of co-variance (ANCOVA) was used to investigate changes between pre and post intervention scores on the total scales of BSI. Table 3 has shown test of between-subject effects. The results show significant difference between the all subtests, that is, the changes were found in posttests was not due to confounding effects of pretest assessments. F index in all subtests are significant (P < 0.01). In follow up analysis (post hoc), the Scheffe test was applied and demonstrate, no significant difference between MBCT and CBT group; But there was a significant difference with TAU except aggression and psychosis.

**Table 3.** Test of Between-subject Effects

source	Sum of squares	df	Mean of squares	F
**GSI**	14.551	2	7.275	41.082 ^a^
**Somatization**	20.249	2	10.125	28.336 ^a^
**OCD**	19.165	2	9.583	23.493 ^a^
**Interpersonal sensitivity**	27.089	2	13.545	36.434 ^a^
**Depression**	26.931	2	13.465	29.143 ^a^
**Anxiety**	12.400	2	6.200	13.785 ^a^
**Aggression**	15.024	2	7.512	22.441 ^a^
**Phobia**	25.340	2	12.670	41.481 ^a^
**Paranoia**	15.392	2	7.696	20.241 ^a^
**Psychosis**	7.185	2	3.592	9.124 ^a^

^a^ P < 0.01

## 5. Discussion

The aim of the present study was to examine efficiency of MBCT in psychiatric symptoms and well-being of patients. Therefore we compared this program with CBT and patients who continued with TAU. It is important to note that MBCT was specially designed for remitted patients with depression. We redesigned combination of MBCT & CBT for more efficiency and application. At the conclusion of that intervention, the participants reported significantly reduced perceived stress and positive status of mind, compared to baseline, similar to the findings of earlier studies (13, 14, 25). From baseline to post- intervention, there was also an improvement in the non-judgmental awareness of participants (i.e. mindfulness self-efficacy). As hypothesized, the practice of mindfulness meditation may have helped study participants to reduce their perception of stress, maintain non-judgmental awareness during different situation, and experience higher levels of healthy state of mind, and significant low level symptoms.

On the other hand, the similar results were found in CBT group, that is, the CBT program associated with reductions in psychiatrics symptoms, and there were no significant difference between two programs. Further, the results suggest that significant reduction in symptoms between earlier groups and TAU, except psychosis scale in BSI. Means and standard deviations of every group have shown significant reduction (Table 2), that is effects of MBCT, CBT, and TAU (medication) is the same. Psychosis scale of BSI has severe deviant thoughts and behaviors statements, may be medication therapy is more effective for severe states of well-being than mild symptoms. Kaplan & Sadock have suggested the same idea (26). The outcomes in the present study were comparable to these previous reports (27, 28). How are the present findings to be explained? The aim of CBT is usually seen as reducing dysfunctional assumptions, and behavioral enhancement, also in MBCT a Meta -cognitive monitoring (29) in relation to the products of dysfunctional schemas is developed. It is noteworthy that a key therapeutic strategy of therapy for psychiatric patients is meta-cognitive skills, that is, to “re-think” their (dysfunctional) thoughts (30). On the other hand MBCT not emphasize on behavioral enhancement, therefore combination of meta- cognitive skills and behavioral components, increase quality of therapy, and similar outcomes.

The majority of the participants of MBCT program found that program was acceptable, enjoyable and beneficial. However most of the group also felt the course was too short and thought that some form of follow up was essential. For many of the participants, being in group was an important normalizing and validating experience. Analysis of the interviews suggests a correlation between the amount of effort participants invested in developing their own mindfulness practice and improvements in psychological well-being. This is in keeping with previous findings that suggest strong links between consistent practice (therapy “homework”) and the process of change (31). The current study was designed to investigate the effects of MBCT techniques for groups, on psychiatric symptoms. Participants were gathered by clinics, and counseling centers. Measures of the outcomes were derived from self-reports and interview administered before and following the 8-week intervention.
